# Advancing Competency-Based Health Professions Education: Insights for Implementation

**DOI:** 10.5334/pme.3021

**Published:** 2026-09-10

**Authors:** Daniel J. Schumacher, Marije P. Hennus, Christy K. Boscardin, Aleda M. H. Chen

**Affiliations:** 1 Cincinnati Children’s Hospital Medical Center, Cincinnati, Ohio, USA; 2 Wilhelmina Children’s Hospital, University Medical Center Utrecht, Utrecht, the Netherlands; 3 Department of Medicine and Department of Anesthesia, University of California San Francisco, San Francisco, California, USA; 4 University of Arkansas for Medical Sciences College of Pharmacy, Little Rock, Arkansas, USA

## Abstract

This Foreword introduces the Advancing Competency-Based Health Professions Education (CBHPE) collection of papers. These articles seek to synthesizing practical guidance for CBHPE implementation at a time when CBHPE is rapidly expanding across the globe. Together, the papers in this series highlight that meaningful CBHPE implementation requires attention to adaptive change, fidelity to core components, relational work among learners and supervisors, responsiveness to local context, and systematic study of implementation processes and outcomes.

In 2024, Schumacher and colleagues asserted the following regarding the current state of competency-based health professions education (CBHPE):

Perhaps the biggest challenge facing [CBHPE] is meaningful implementation as intended. Van Melle and colleagues have published five core components of [CBHPE] implementation, which provides a “North Star.” [1] However, available time and resources may too often determine implementation success…[making] both compassion and collaboration…foundational to overcoming the implementation challenges that [CBHPE] faces. As [CBHPE] implementation efforts expand logarithmically, they do so in a world and healthcare milieu that is increasingly despondent, on edge, and even angry. In this milieu, compassion is necessary to demonstrate sincere understanding for barriers that are not personal but rather systemic. This positions those meeting with success to more meaningfully collaborate with those who are not yet [2].

This sentiment deftly captures the spirit and intent of a series of papers from members of the International Competency-based Health Professions Educators Collaborative (ICBHPE) that follow this Foreword. As leaders in CBHPE globally, the Collaborative has extensive experience with successful CBHPE implementation efforts but also lived experiences with the myriad challenges to implementing and sustaining CBHPE. These challenges have led to searing punditry from authors who have rebuked CBHPE as “faith-based medical education” [3], a “discourse of infalliability” [4], and “monkey see, monkey do.” [5] While these critiques may not reflect the intentions of CBHPE proponents, they resonate with challenges many educators have experienced when implementation has been incomplete, superficial, or poorly supported. These critiques also underscore that the central question is no longer whether CBHPE is conceptually appealing, but whether it can be implemented with sufficient fidelity, support, and responsiveness to context to achieve its intended aims.

Spearheading dialogue to overcome CBHPE implementation issues became the driving theme at a February 2025 summit of international members of the ICBHPE. As global scholars seeking to advance CBHPE, synthesizing practical guidance for CBHPE implementation at a time when CBHPE was rapidly expanding across the globe seemed especially timely. The papers that follow this Foreword seek to achieve this goal. Together, the papers in this series highlight that meaningful CBHPE implementation requires more than adopting new assessment practices or invoking competency-based language. It requires attention to adaptive change, fidelity to core components, relational work among learners and supervisors, responsiveness to local context, and systematic study of implementation processes and outcomes. This Foreword introduces these cross-cutting themes and situates the papers as a coherent implementation agenda that advances practical, context-sensitive guidance for CBHPE implementation.

## Implementing CBHPE is an Adaptive Challenge

In our view, CBHPE implementation is an adaptive challenge, not a technical problem [6,7] as highlighted by the papers in the series [8,9,10,11,12,13,14,15,16,17,18,19,20,21]. A technical problem is one in which the problem and solution are clear, the work will be managed by an expert, and the focus is on expediting a fix. A broken pipe in your house is a technical problem. Mending or replacing the pipe is the solution, it will be done by a plumber, and you will seek to have it done as quickly as possible to restore water supply to your house. In comparison, an adaptive challenge does not present a clear problem or solution [6,7]. Solving adaptive challenges requires engaging with stakeholders in addressing the challenges posed by changes in beliefs, roles, relationships and identifying potential solutions. Thus, CBHPE implementation cannot be understood apart from the context in which it occurs. Framing CBHPE implementation as an adaptive challenge shifts attention from simply adopting CBHPE as a framework to understanding the local conditions, relationships, resources, and values that drive the implementation success. Papers from Hall et al [13] and Englander et al [8] detail successful efforts in implementing and advancing CBHPE and the importance of learning from and with local context and stakeholders. In an effort to better learn from CBHPE implementation experiences such as those shared in these papers, the CBHPE Outcomes Theory of Change and Reporting Guidelines [11,12] emphasize documenting implementation fidelity, contextual influences, and local adaptations so that educators can better understand what works, for whom, and under what conditions.

Given the characteristics of adaptive challenges (hard work, work to be avoided), it is perhaps not surprising that implementing CBHPE is often treated as a technical problem (limited work, easy fix) in efforts to implement it. For example, some may view AI as an “easy fix” to problems encountered in CBHPE implementation. However, the emergence of AI does not transform CBHPE implementation into a technical problem. Rather, as highlighted by Lomis et al [18], successful use of AI depends on navigating complex interactions among technology, people, workflows, incentives, and organizational culture. AI is only a tool, and its potential impact lies in how individuals and organizations adapt it, further illustrating the adaptive nature of CBHPE implementation.

CBHPE implementation is further complicated by a common feature of adaptive challenges: the benefits are broadly distributed, while the investments of time, expertise, and resources are often concentrated among those responsible for implementation [17]. As a result, those who bear the burden may not be those who receive immediate benefits, creating challenges and opportunities which are noted by Rhoney and colleagues [17]. Further, selection systems that reward activities misaligned with competency development further illustrate how CBHPE implementation is shaped by broader educational structures beyond the curriculum itself [21]. A common challenge runs across these considerations: implementing CBHPE requires balancing fidelity to its core principles with thoughtful adaptation to local contexts, cultures, and constraints.

## CBHPE Core Components: CBHPE is an Approach to Education, Not Simply a New Assessment Strategy

In 2019, van Melle and colleagues published a seminal paper detailing the core components of CBHPE [1]; in other words, the things you must do to say that you have implemented CBHPE. The core components are: 1) outcome-based competencies that define the goals of education, 2) educational experiences focused on achieving the defined outcomes, 3) sequenced progression through training based on development, 4) individualized learning experiences to support growth, and 5) programmatic assessment. Although only one of these five core components focusses on assessment, many “CBHPE implementation” reports found in the literature focus entirely on assessment. Fidelity of implementation is foundational to saying you have “implemented CBHPE.” This distinction matters because implementation efforts that focus narrowly on assessment may adopt some of the language of CBHPE while leaving the broader educational system largely unchanged. Therefore, almost all papers in the series include a focus on, and are often framed around, van Melle’s CBHPE core components [8,9,10,11,12,13,14,15,16,17,18,19,20]. Furthermore, all papers in this series [8,9,10,11,12,13,14,15,16,17,18,19,20,21] collectively illustrate that successful CBHPE implementation requires attention to far more than assessment alone and depends on alignment across the core components described by van Melle and colleagues, of which only one focuses on assessment.

## The Relationship Between Trainees and Supervisors

Given that the CBHPE core components focus on promoting and supporting trainee development, the relationship between trainee (to be developed) and supervisor (to support development) is foundational. Multiple papers in the series highlight that building trust between trainees and supervisors is essential [9,14,15,16,18]. Coaching is a hallmark of CBHPE [8,9,11,12,15,16,20]. As we seek to highlight the importance of coaching and as AI is evaluated to aid in coaching, AI should complement rather than replace the human relationships, coaching, and professional judgment that underpin CBHPE [18]. This relational foundation also clarifies the role of technology such as AI – tools may support documentation, pattern recognition, feedback, or coaching processes, but they cannot substitute for the trust and professional judgment required for meaningful learner development. Further, implementing and sustaining CBHPE must be done with trainees [11,12,16]. Centering on relationships, implementation efforts must evaluate equity and elevate and value historically marginalized voices. As highlighted by papers led by Ahn, Busari, and Chen, meaningful implementation requires attention to whose knowledge, experiences, and priorities shape CBHPE in a given setting [10,11,12,20].

As Turner and others argue [10,11,12,16], CBHPE implementation should be adapted to local needs, contexts, and purposes rather than assuming a single model, largely generated by the Global North, will translate across environments. At the same time, achieving the promise of CBHPE requires acknowledging and valuing the substantial efforts of faculty and supervisors who undertake the complex work of assessment, coaching, and program implementation [9,16,17]. Sustaining CBHPE depends not only on supporting learners, but also on supporting the educators whose work makes learner development possible. Faculty and supervisors are not merely implementers of CBHPE; they are central participants, with trainees, in the adaptive work required to make CBHPE credible, educationally meaningful, and sustainable.

## Toward a Science of CBHPE Implementation

Several papers in this series focus not only on how to implement CBHPE but also on learning from implementation processes [8,11,12,13]. Achieving this goal requires a discussion of how CBHPE should work as well as transparent reporting of interventions, implementation processes, contextual factors, and outcomes so that the field can accumulate actionable knowledge across settings. By systematically studying implementation, educators can distinguish between shortcomings in CBHPE as an educational approach to reflecting on how it has been implemented, adapted, or sustained. In doing so, implementation science provides a pathway away from the critiques of CBHPE as “faith-based medical education” and toward a cumulative science of educational change. Implementation science provides a way to move beyond isolated success stories or failed implementations by providing a framework for asking how, why, for whom, and under what conditions CBHPE produces desired outcomes. Rather than assert CBHPE will succeed because its principles are compelling, the field must continuously test, refine, and learn from implementation efforts across diverse settings. The papers in this series represent an important step in that direction.

## Conclusion

Taken together, the papers in this series suggest that the success of CBHPE depends less on the framework itself and more on how it is enacted within educational systems. Competencies, assessments, coaching models, reporting frameworks, and technological innovations provide important structures, but none function independently of the people, relationships, cultures, and organizations in which they are embedded. Across diverse contexts, the papers in this series illustrate that meaningful implementation requires attention not only to what CBHPE is but also to how it is operationalized, supported, and sustained. Viewed collectively, these contributions move the conversation beyond whether CBHPE works toward a more important question: how can CBHPE be implemented in ways that realize its intended benefits for learners, educators, patients, and health systems? In doing so, the contributions in this series provide practical guidance for educators while contributing to a growing science of CBHPE implementation.
